# Compressing Experiences of Optical Resolution Trials, Based on Diastereomeric Salt or Co-Crystal Formation, into Ternary Equilibrium Melting Phase Diagrams of Two Chiral Enantiomers and a Resolving Agent Molecule with the Help of DSC and Powder XRD

**DOI:** 10.3390/molecules31040623

**Published:** 2026-02-11

**Authors:** János Madarász

**Affiliations:** Department of Inorganic and Analytical Chemistry, Faculty of Chemical Technology and Biotechnology, Budapest University of Technology and Economics, Műegyetem rkp. 3, H-1111 Budapest, Hungary; madarasz.janos@vbk.bme.hu

**Keywords:** optical resolutions, crystalline racemates, enantiomers, and diastereomeric species, chiral resolving agents, eutectic relations and melting processes, binary and ternary phase diagrams, melting temperature and enthalpy of fusion, DSC, estimation of liquidus curves, eutectic compositions and temperatures, Fogassy’s parameter of resolubility, unit cell indexing, DASH modeling, powder XRD patterns

## Abstract

This study contains a combination of a review and a related individual case study, which discusses the possibility of predicting the success of enantiomers’ optical separation using fractional crystallization of diastereomeric salts. The key idea is to use relatively simple and rapid experimental methods, such as differential scanning calorimetry (DSC) and powder X-ray diffraction (XRD), to construct ternary melting diagrams. These diagrams can be used for visualization and predicting compositional conditions favorable for successful separation. The main limitations are also mentioned, such as the ideal eutectic behavior of components and the need to identify all crystalline phases in the system. For demonstration, three novel studies, attempts in ternary resolution systems of racemic *o*- and *p*-chloromandelic acids with chiral 1-cyclohexylethylamine or pregabalin, resulting in either declined or promising aspects for a successful resolution, were completed, and the corresponding individual ternary phase diagrams have been compiled and presented, as well. In addition, indexing and modeling of one of the diastereomeric salts’ unit cells have been successfully carried out by means of powder X-ray diffraction, using the DASH software package.

## 1. Introduction

The formation and fractionated crystallization of diastereomeric salts or co-crystals are one of the most frequently applied and probably one of the simplest methods for the optical resolution of binary mixtures of enantiomeric molecules with mirror images [1] (pp. 39–41). In general, binary mixtures of crystals, based on the extent of their miscibility in the solid phase, can be classified into three fundamental types: (a) a physical mixture, i.e., conglomerate of crystals showing eutectic relation (if components are fully immiscible in crystalline state); (b) at least one new common crystalline addition compound with a definite stoichiometric ratio, which is usually in eutectic relation with the parent crystalline phases (as case of partial miscibility of components in solid phase); and (c) a solid solution (components which are fully miscible in any ratio in crystalline state). These cases for solid molecular pairs can be characterized and represented by means of binary (melting) phase diagrams built upon a series of differential scanning calorimetric (DSC) measurements. Such classifications based on types of fusion diagrams are well known among racemic binary compositions [1] (p. 32) [2]. The liquidus curve of melting phase diagrams can be modeled using simplified Schröder–van Laar or Prigogine–Defay equations in the case of eutectic crystal conglomerates or racemic compounds of enantiomers, respectively [1] (pp. 46–47, 88–91) [2], using only the individually measured melting points (*T*^f^_i_ in K) and molar enthalpy values of fusion (Δ*H*^f^_i_, J/mol) of pure crystalline enantiomeric components.

The most frequently applied method in optical resolutions is trying to utilize an expected difference in the solubility of various salts or co-crystals as diastereomeric solid crystalline species, which could be formed from at least one of the two mirror enantiomers to be resolved with a chosen chiral agent, when all of them are dissolved in a certain solvent, at a certain temperature. Anyhow, an opportunity for the successful fractionated crystallization of the corresponding diastereomeric species, intended to achieve, is not guaranteed, especially when the difference in solubility is small. A rapid estimation method to compare the otherwise unknown solubility of various crystalline solids could be established by collecting corroborating thermal stability judgments on their crystal structures, based on checking their crystallinity via powder X-ray diffraction (XRD) and their melting temperature and enthalpy of fusion. The data for the latter two can usually be measured and collected for all the crystalline solids occurring in the ternary system of the two enantiomers and resolving chiral agent via differential scanning calorimetry (DSC). The collected data can be compressed into ternary equilibrium melting phase diagrams, assuming eutectic relations (validity of simplified Schröder–van Laar equation) among the crystalline species present in the system. Such constructed triangular diagrams, common in physical chemistry, could reflect and describe a potential compositional region of success for efficient resolutions.

When applying a resolving agent, a third component inevitably occurs in the racemic resolution system; at least ternary fusion phase diagrams are also needed to describe and represent the detailed complexity of the whole system, beyond the pairwise obtainable binary ones [3]. A triangular fusion phase diagram of the two mirror enantiomers, together with the chosen resolving agent, is usually composed of joining several three-component fusion phase (sub)diagrams of three-component (eutectic) immiscible crystalline phases [4], as a result of the occurrence of one, two, or more crystalline diastereomeric phases in the resolution system.

When both the expected diastereomeric salt species can potentially occur within the resolution system at the same time, it was recognized by Kozma et al. [5] that the so-called *eutonic* solubility concentration ratio of the two diastereomeric salts in the system, usually tediously measurable in the liquid phase, nevertheless, can reliably be estimated with the *eutectic* composition of the corresponding binary solid–melt phase diagram, via a relatively simple calculation for the latter. So, if the two pure salts can be prepared separately and a eutectic relation can be assumed between them, only a few DSC measurements are needed to obtain their melting point and enthalpy of fusion, in order to calculate their eutectic composition, which can provide a reliable guess for their mutual solubility relation and expected behavior during resolution via fractionated crystallization [6].

The lucky cases, when only one of the expected diastereomeric salts or co-crystals precipitates (first) from the resolution liquor, or oppositely, the unfortunate cases, when DSC measurements cannot provide us with either melting points or entalphy of fusion, all are discussed in ref. [7]. There, prof. Fogassy and his coworkers concluded the main ‘rules’ of a successful resolution based on their 16-case study, summarizing that “ … during optical resolution via fractional crystallization of diastereomeric salt pairs always that diastereoisomeric salt precipitates (first), which has the higher melting point. When one of the salts is amorphous, that remains in the mother liquor. If one of the diastereoisomeric salts crystallizes with solvate, that will precipitate during optical resolutions.” [7].

**Figure 1:**
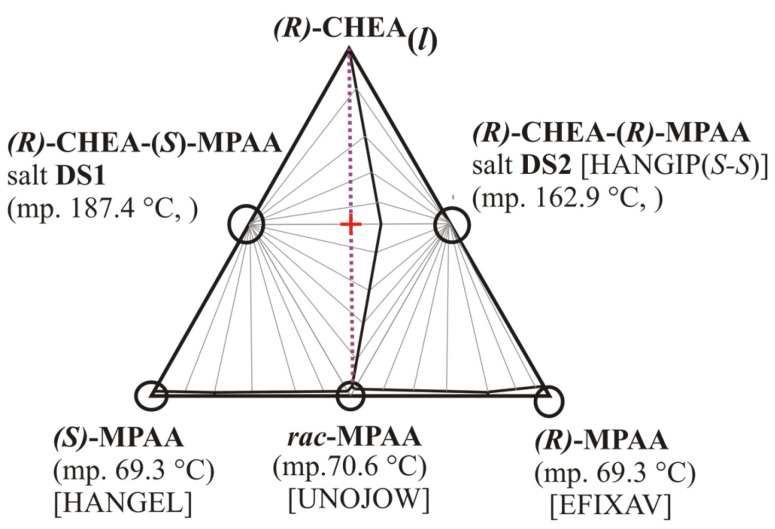
Calculated schematic three-component melting phase diagram for α-methoxyphenyl- acetic acids (**MPAA**) and (*R*)-1-cyclohexylethylamine (**CHEA**), redrawn from ref. [8] (Figure No. 7 therein, with courtesy). The five solid crystalline components of eutectic relations, occurring in the ternary system are marked by circles. {Note: CSD (Cambridge Structural Database [9]) refcode is HANGEL [8], EFIXAV [10], and UNOJOW [11] for *S*-, *R*-, and racemic **MPAA,** respectively}. **DS1** and **DS2** correspond to the expected two diastereomeric salt species, which can be formed between chiral α-methoxyphenylacetic acids and (*R*)-1-cyclohexylethylamine (in 1:1 molar ratio). The two diastereomeric crystalline salts of eutectic relation are the dominating solids in large compositional domains limited by the thich liquidus lines in the triangle, where the **DS1** crystalline salt has a higher melting point and lower solubility then **DS2** salt does. {Note: A CSD (Cambridge Structural Database [9]) refcode is HANGIP for mirror *S*-*S*-salt, [8]}. The difference in their melting points is ca. 25 °C. Dotted long magenta line sequence corresponds to wide variety of promising mixing ratios between the crystalline ***rac*-MPAA** and the liquid (*R*)-1-cyclohexylethylamine to achieve a successful resolution, meanwhile the red cross indicates a usual test composition point, 1:1 molar ratio of racemate to resolving agent.

For cases when both the expected diastereomeric species can occur in crystalline form, prof. Fogassy and his coworkers also stated that “Efficient resolution can be expected, if there are at least 20 K differences between the melting points of the diastereoisomeric salt pair.” [7]. Such a lucky case was also recently demonstrated by DSC measurements on pure salts and subsequent construction of ternary phase diagrams for a resolution system of racemic 2-methoxy-2-phenylacetic acids with chiral 1-cyclohexylethylamine [8], when a ca. 25 °C melting point difference for diastereomeric salt species was observed (Figure 1), and the diastereomeric salts’ eutectic molar composition was calculated as x_eu_ = 0.30 (of favoring salt No. 4), while the corresponding ‘resolubility value’ (named also as Fogassy’s parameter) was estimated to be F_max_ = 2 − 1/(1 − x_eu_) = 0.57, as a measure of the theoretical maximum efficiency of the resolution obtainable in the given ternary system of racemic acids and chosen resolving chiral amine [8].

**Figure 2:**
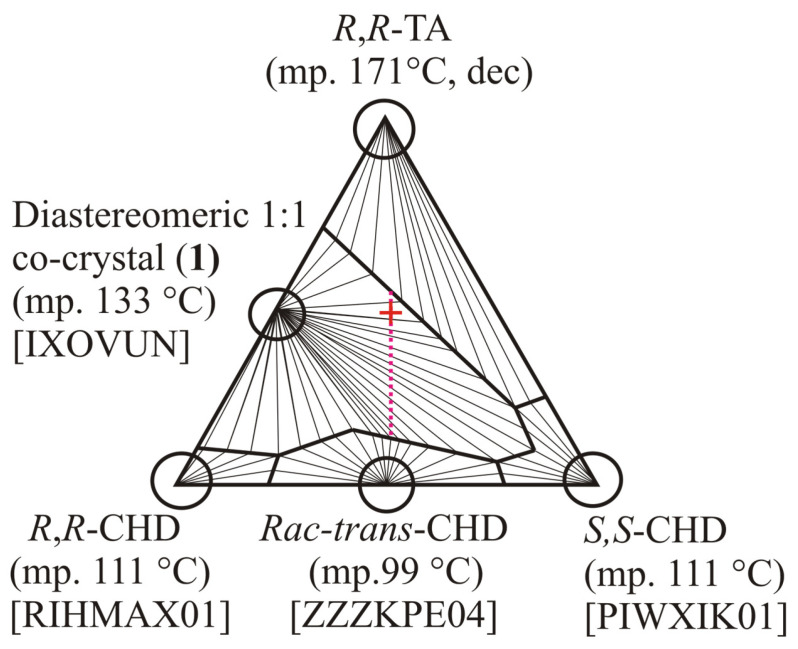
Calculated informative three-component melting phase diagram for chiral *trans*-1,2-cyclohexanediols (**CHD**) to be resolved using chiral (*R*,*R*)-tartaric acid (**TA**), redrawn from ref. [13] (Figure No. 9 therein, with courtesy). The five solid crystalline components of eutectic relations, occurring in the ternary system are marked by circles. {Note: CSD (Cambridge Structural Database [9]) refcode is RIHMAX01 [3], PIWXIK01 [14], and ZZZKPE04 [3] for chiral *R*,*R*-, *S*,*S*-, and racemic **CHD**, respectively}. Liquidus lines (thick lines within the triangle) are limiting the compositional regions, in which a circled crystalline phase is a dominating solid as of the highest melting point and the lowest solubility. Here, compound No. **1** (CSD refcode IXOVUN, [13]) corresponds to the only existing crystalline diastereomeric species, a diastereomeric co-crystal between chiral (*R*,*R*)-1,2-cyclohexanediol and (*R*,*R*)-tartaric acid (in 1:1 molar ratio) in the resolution system. [No more crystalline phase built up from both (*S*,*S*)-1,2-cyclohexanediol and (*R*,*R*)-tartaric acid is known]. Dotted lilac line sequence corresponds to various recommended mixing ratios between *rac*-*trans*-CHD racemate compound and (*R*,*R*)-tartaric acid to achieve a successful resolution, while the red cross on it is the common test composition point, 1:1 molar ratio of racemate to resolving agent.

Some more favorable cases, when one member of the salt pair is amorphous, which does not form any crystals and remains in the mother liquor, were also demonstrated recently by Faigl et al. [12] and Thorey et al. [13] (Figure 2). Generally, the larger the asymmetry in the ternary molar melting phase diagram, the better resolution results can be expected, at least in thermodynamically controlled (equilibrium) resolution ways.

Here, three novel case studies, in the proposed ternary resolution systems of racemic *o*- and *p*-chloromandelic acids with chiral 1-cyclohexylethylamine or pregabalin, with either promising or declined aspects, are presented, through calculation and construction of the corresponding and representative ternary melting phase diagrams.

## 2. Results and Discussion

### 2.1. Construction of Ternary (Triangular) Phase Diagrams of p- and o-Chloromandelic Acids with Chiral 1-Cyclohexylethylamines; Extraordinary Occurrence of the ‘Mixed Diastereomeric Salt’, Double Salt of Diastereomeric Salt Pair (Case Nos. 1 and 2)

**Figure 3:**
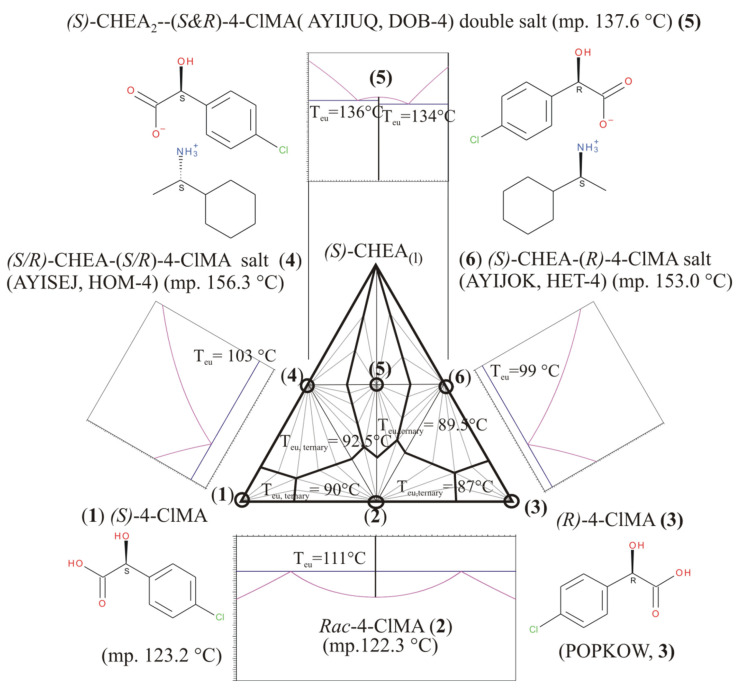
Constructed schematic overall ternary and some of the binary phase diagrams in (*S*)-4-chloromandelic acid (**1**)—(*R*)-4-chloromandelic acid (**3**)—(*S*)-1-cyclohexylethylamine three-component system (Case No. 1). {Note: CSD (Cambridge Structural Database [9]) refcode of (*R*)-4-chloromandelic acid is POPKOW [17]} Equilibrium calculations are based on the corresponding data from ref. nos. [15,16] and private communication to JM. Subternary systems are also defined by triplets of components, concerning all the solid crystalline compounds (circled), including chiral and racemic 4-chloromandelic acids and three supramolecular salts (Salt Nos. **4**, **5**, and **6** with CSD reference codes AYISEJ, AYIJUQ, AYIJOK, respectively), which are co-crystallized compounds of the chiral 4-chloromandelic acids and 1-cyclohexylethylamines [16]. The overall three-component temperature vs. molar fraction (*T* − *x*) phase diagram has been assembled from ternary subdiagram of crystalline phase relations, namely assuming eutectic behavior among all the crystalline solids (circled) and validity of Schröder–van Laar equations describing liquidus curves. The bottom binary phase diagram of 4-chloromandelic acid system corresponds to that of given in [15]. Estimation of the joint binary eutectic phase diagram (top) of Salt Nos. **4**, **5** and **6**, and the calculations of liquidus curves around double Salt No. **5** are carried out—formally—by application of Prigogine–Defay equation, as well, and it also corresponds to that of given in electronic supporting information part of Ref. [16] (ESI therein). The usual test composition point (1:1 molar ratio of racemate to be resolved to the resolving agent) is sitting on the middle of the horizontal line between the wanted diastereomeric salt pair, exactly where their ‘addition compound’, **5** is also located as dominating solid crystalline species.

**Figure 4:**
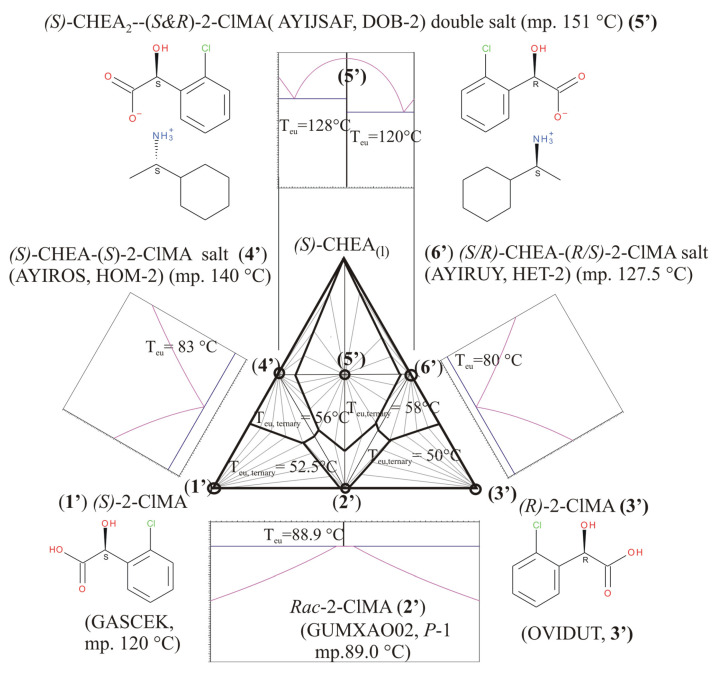
Constructed schematic overall ternary and some of the binary phase diagrams in (*S*)-2-chloromandelic acid (**1′**)—(*R*)-2-chloromandelic acid (**3′**)—(*S*)-1-cyclohexylethylamine three-component system (Case No. 2). {Note: CSD (Cambridge Structural Database [9]) refcode is GASCEK [18], OVIDUT [19], and GUMXAO02 [20] for (*S*)-, (*R*)-, and (*RS*)-2-chloromandelic, respectively.} Equilibrium calculations are based on the corresponding data from ref. nos. [15,16] and private communication to JM. Subternary systems are also defined by triplets of components, concerning all the solid crystalline compounds (circled) including the two chiral and the racemic 2-chloromandelic acid (**2′**) and three supramolecular salts (Salt Nos. **4′**, **5′**, and **6′** with CSD reference codes AYIROS, AYISAF, AYIRUY, respectively), which are co-crystallized compounds of the chiral 2-chloromandelic acids and 1-cyclohexylethylamines [16]. The overall three-component temperature—molar fraction (*T* − *x*) phase diagram has been assembled from ternary subdiagram of crystalline phase-relations assuming eutectic behavior among all the three crystalline solids and validity of Schröder–van Laar equations describing liquidus curves. The bottom binary phase diagram of 2-chloromandelic acid system corresponds to that of given in ref. [15]. (The liquidus curves in racemic region are so flat, that no distinction between the solidus and liquidus curves can be observed as shown in ref. [15]). Estimation of the joint binary eutectic phase diagram (at the top) of Salt Nos. **4′**, **5′**, and **6′**, and the calculations of liquidus curves around double Salt No. **5′** are carried out—formally—by application of Prigogine–Defay equation, as well, and it also corresponds to that of given in ESI part of Ref. [16]. The usual test composition point (1:1 molar ratio of racemate to be resolved to the resolving agent) is sitting on the middle of the horizontal line between the wanted diastereomeric salt pair, exactly where their ‘addition compound’, **5′** is also located as dominating solid crystalline species.

Recently, Kőrösi et al. [15] reported the binary equilibrium melting phase diagram of *o*-, *p*-, and even *m*-chloromandelic acids, in which chiral enantiomeric species form pairwise racemates (i.e., racemate molecular compounds). Furthermore, Bereczki et al. [16] intended to resolve both the racemic compounds of *rac*-*o*- and *rac*-*p*-chloromandelic acids with the help of chiral (*S*)-1-cyclohexylethylamines and carried out a preliminary screening for diastereomeric salts in the given ternary systems. They found in both investigated systems that there were surprisingly three ‘diastereomeric salts’ in each one, i.e., both systems could be described as having an extra salt, so-called ‘double salt’ (DOB-4, DOB-2, respectively), existing beyond the expected salt pair, and corresponding to a crystalline addition compound of the two classical diastereomeric members. All the crystal structures of 1-cyclohexylethylammonium 2-chloromandelate (***S*-*S***, ***R*-*S***, and ***SS*-*SR***) and 1-cyclohexylethylammonium 4-chloromandelate (***R*-*R***, ***S*-*R***, and ***SS*-*SR***) salt were determined via single-crystal X-ray diffraction [16], and all of them are now already included in the Cambridge Structural Database (CSD) [9]. At the same time, they also observed a very-very low or lack of efficiency in the attempted optical resolutions, which seemed to be associated with the formation of these ‘double salts’ during the optical resolution experiments, starting with 1:1 molar ratios of racemic composition to the resolving agent, as well. Beyond the individual crystal structures, the melting points and enthalpy of fusion of all of the 2 × 3 = 6 ‘diastereomers’ (including both the diastereomer pairs and double salt) were measured using differential scanning calorimetry [16]. Using their published thermal fusion data (Table 1), the corresponding ternary phase diagrams have now been constructed by us and are shown in Figure 3 and Figure 4.

Actually, these two diagrams demonstrate that the usual test composition point (1:1 molar ratio of racemate to be resolved to the resolving agent) is sitting in the middle of the horizontal line between the wanted diastereomeric salt pair, in the same place exactly where their ‘addition compounds’, **5** or **5′,** are also located as dominating solid crystalline species, respectively. Thus, when we try to dissolve a racemic composition with an equivalent molar amount of the said resolving agent together, - from which solution a physical mixture of diastereomeric species (**4**, **6** or **4′**, **6′**, respectively) of different solubility is expected to obtain, - only their addition compounds (**5** or **5′**) could be formed without providing any resolution. Furthermore, we can see, when we applying the chosen resolving agent (chiral (*S*)-1-cyclohexylethylamines), the whole triangular diagram of both resolution systems is almost symmetrical for the vertical median (Figure 3 and Figure 4).

### 2.2. Construction of Ternary Phase Diagrams of o-Chloromandelates with Chiral Pregabalin (Case No. 3)

#### 2.2.1. DSC Measurements and the Corresponding Triangular Phase Diagram

**Figure 5:**
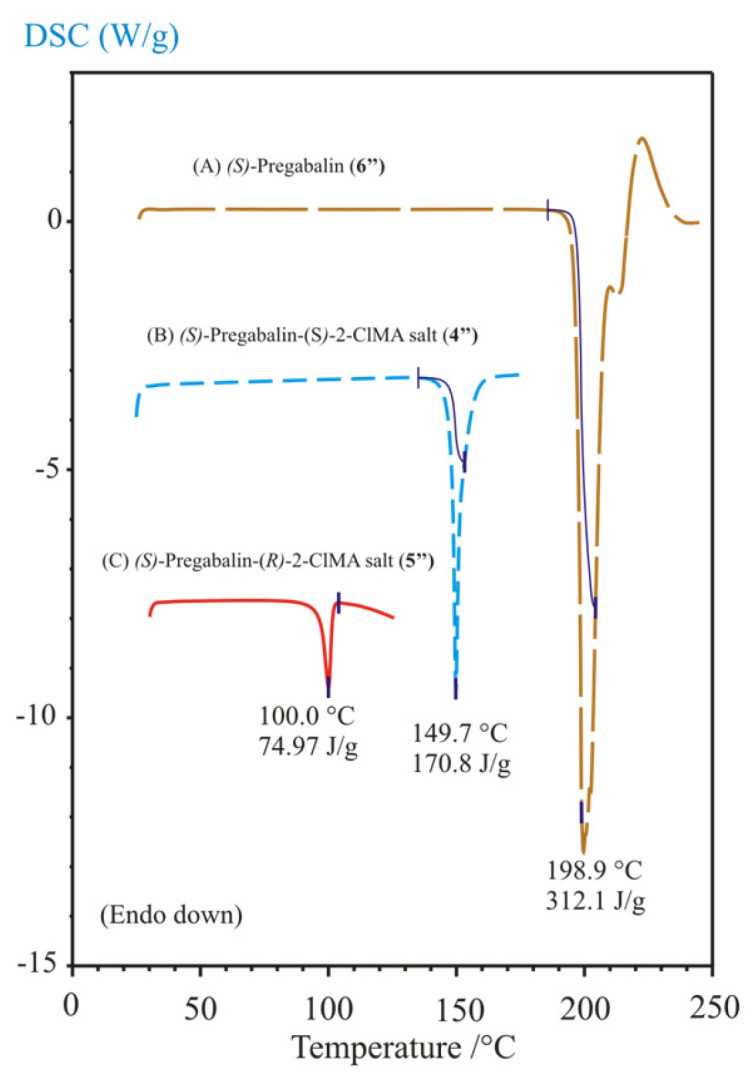
Differential scanning calorimetric (DSC) curves of the chiral resolving agent, (*S*)-pregabalin (**6″**, A, top, brown curve) and the pure diastereomeric salts, (*S*)-pregabalin-(*S*)-2-chloromandelic acid salt (**4″**, B, middle, blue curve), and (*S*)-pregabalin-(*R*)-2-chloromandelic acid salt (**5″**, C, bottom, red curve) with the observed melting points, which are 199, 150, and 100 °C, respectively. The enthalpy of (*S*)-pregabalin and *S-S*-salt is corrected for their decomposition process, which seems to escort the fusion itself. (Latter substances might melt with decomposition).

**Figure 6:**
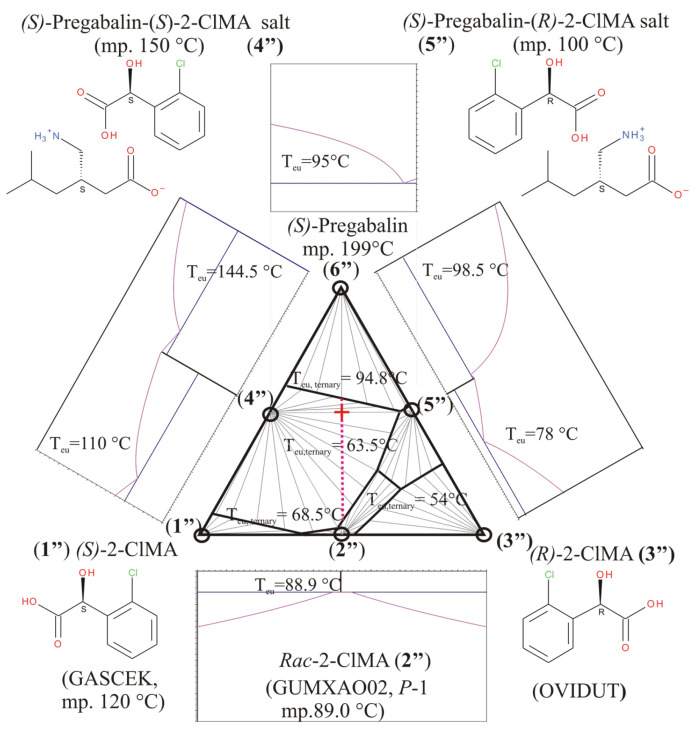
Constructed schematic overall ternary and some of the binary phase diagrams in (*S*)-2-chloromandelic acid (**1″**)—(*R*)-2-chloromandelic acid (**3″**)—(*S*)-pregabalin (**6″**) three-component system. Equilibrium calculations are based on the corresponding data from ref. nos. [15,22] and private communication to JM. Subternary systems are also defined by triplets of components, concerning all the solid crystalline compounds (circled), including chiral and racemic 2-chloromandelic acids and two supramolecular salts (Salt Nos. **4″** and **5″**), which are distinct co-crystallized compounds of the enantiomeric 2-chloromandelic acids and (*S*)-pregabalin [22]. The overall three-component temperature—molar fraction (*T* − *x*) phase diagram has been assembled from ternary subdiagram of crystalline phase-relations assuming eutectic behavior among all the three crystalline solids and validity of Schröder–van Laar equations describing liquidus curves (Calculated T_eu, ternary_ ternary eutectic temperatures are also shown). The bottom binary phase diagram of 2-chloromandelic acid system corresponds to that in [15] (The liquidus curves in racemic region are so flat, that no distinction between the solidus and liquidus curves can be observed as shown in ref. [15]). Estimation of the joint binary eutectic phase diagram (at the top) of Salt Nos. **4″** and **5″**, and the calculations of liquidus curves are carried out by application of Schröder–van Laar equation, as well, and it corresponds to that of given in [22]. Dotted lilac line sequence (with the red cross on it as common test point) corresponds to various recommended initial mixing ratios between *rac*-2-chloromandelic acid and (*S*)-pregabalin to achieve a successful resolution.

Nevertheless, Szeleczky et al. [21] reported the successful resolution of racemic *o*-chloromandelic acid with chiral pregabalin [(*R*)-3-isobutyl-γ-aminobutyric acid], beyond that of racemic mandelic acid. As a continuation of their work, we prepared both diastereomeric salts of *o*-chloromandelic acid with a chiral pregabalin [(*S*)-3-isobutyl-γ-aminobutyric acid] [22]. Furthermore, we measured their melting points and enthalpy of fusion, beyond those of (*S*)-pregabalin, in order to construct the corresponding ternary melting phase diagram of this resolution system and to compare it with the previous one (Figure 4) to explain the achieved resolution of *o*-chloromandelic acids. The differential scanning calorimetric (DSC) curves of the chiral resolving agent and the pure diastereomeric salts (**4″** and **5″**) are shown in Figure 5. The observed melting points are 100, 150, and 199 °C for pure separately prepared *S-R*-salt, *S-S*-salt, and the starting (*S*)-pregabalin, respectively. The enthalpy of (*S*)-pregabalin is corrected for its decomposition process, which seems to escort the fusion itself (i.e., it is a melting process together with decomposition). The corrected values of 75, 171, and 312 J/g were used in the triangular phase diagram calculation (Figure 6) of this system.

During a resolution process, first, the (*S*)-pregabalin-(*S*)-2-chloromandelic acid salt (**4″**) precipitates immediately and it can be filtered; nevertheless, the other diastereomer salt, (*S*)-pregabalin-(*R*)-2-chloromandelic acid salt (**5″**), can also be obtained further on as well-grown crystals from the mother liquor on longer time scale. Because of the large difference (50 °C) between the melting points of the diastereomeric salt pair’s members, the ternary phase diagram obtained (Figure 6) is a very asymmetric one, related to the vertical median line in the middle. The dotted lilac line sequence corresponds to various recommended initial mixing ratios between *rac*-2-chloromandelic acid racemic compound and (*S*)-pregabalin to achieve successful resolutions. The usual test composition point, 1:1 molar ratio of racemate to resolving agent (indicated by a red cross), is within a large favorable domain, where the dominating species is (*S*)-pregabalin-(*S*)-2-chloromandelic acid salt (**4″**) of higher melting point and lower solubility. The diastereomeric salts’ eutectic molar composition is calculated as x_eu_ = 0.086 (of the favored high melting salt **4″**), while the corresponding resolubility value (Fogassy’s parameter) is estimated to be F_max_ = 2 − 1/(1 − x_eu_) = 0.906, as a maximum measure of the theoretical efficiency of resolution obtainable in this system.

#### 2.2.2. Powder XRD Patterns and FTIR Spectra of the Pure Diastereomeric Salts (**4″** and **5″**)

**Figure 7:**
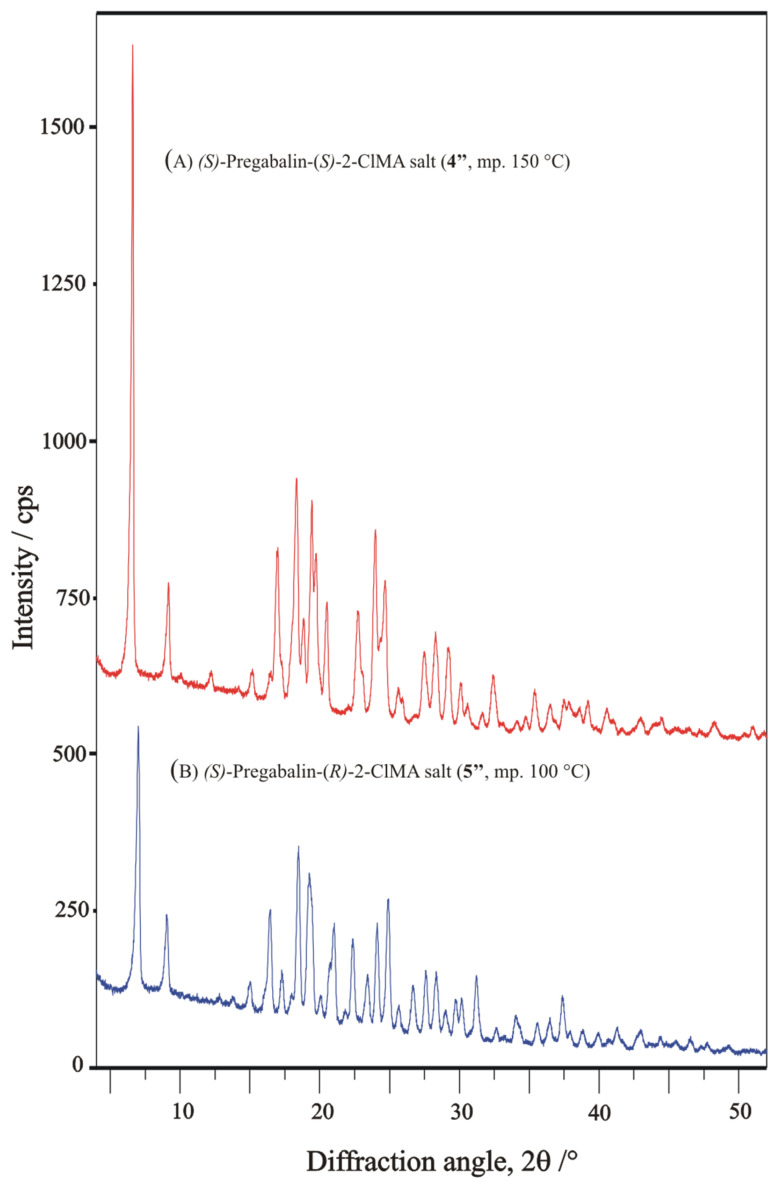
Powder X-ray diffraction (XRD) patterns of the pure diastereomeric salts, (*S*)-pregabalin-(*S*)-2-chloromandelic acid salt ((**A**) **4″**, top) and (*S*)-pregabalin-(*R*)-2-chloromandelic acid salt ((**B**) **5″**, bottom) measured between 2θ = 4–52°.

**Figure 8:**
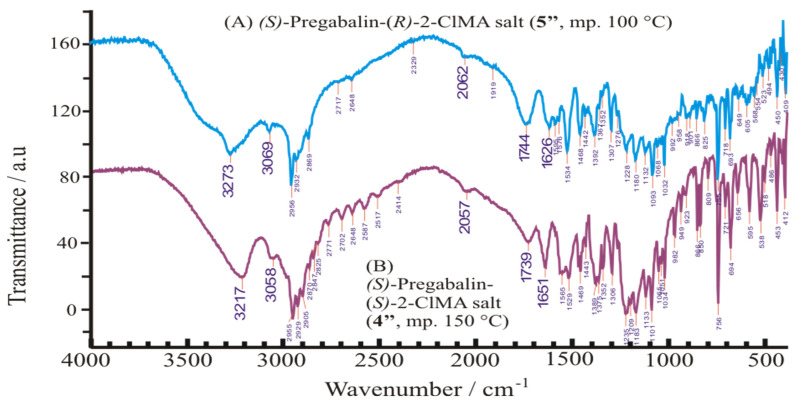
FT-IR spectrum of the pure diastereomeric salts, (*S*)-pregabalin-(*R*)-2-chloromandelic acid salt ((**A**) **5″**, top) and (*S*)-pregabalin-(*S*)-2-chloromandelic acid salt ((**B**) **4″**, bottom) measured between 4000–400 cm^−1^.

Both solid diastereomeric salt-pair samples (**4″** and **5″**) are crystalline, as shown in Figure 7. Despite their patterns look like somewhat similar, nevertheless, they have completely different reflections. Their FT-IR spectra might also seem to be, again, more or less similar; nevertheless, their absorption bands have significantly different positions (Figure 8).

#### 2.2.3. Indexing and Modelling Diastereomeric Salts’ Unit Cell by Means of Powder X-Ray Diffraction, Applying the DASH Software Package (4.0.0 Release) and Crystal Coordinates Coming from Former Single-Crystal X-Ray Structure Determination

From the powder X-ray diffraction patterns exhibited in Figure 7, some structural predictions have been tried to extract for the pure diastereomeric salts with the help of the integrated DASH software package [23], with a versatile, graphical-user-interface-driven computer program, which was earlier distributed within the Cambridge Structural Database System [9] package but is now available separately and publicly at the following link https://github.com/ccdc-opensource/dash/releases, URL (accessed on 1 February 2026). The DASH program package was—among others—originally planned for structure solution [24,25,26,27] from powder diffraction patterns collected in very high resolution, obtainable by synchrotron radiation, especially to determine crystal structures of large-volume commercial pharmaceuticals [28,29] [Note. A large number of additional references should also be mentioned here (but they are not listed) e.g., which are citing this basic ref. [28] and parts of a project of J.A. Kaduk on series of publications to determine the crystal structures of large-volume commercial pharmaceuticals and include high-quality powder diffraction data for them in the Powder Diffraction File, one of the latest articles of his is ref. [29]], but it might be useful for the structural evaluation of powder diffraction profiles collected using laboratory X-ray tubes, as well [30,31].

The XRD pattern of the pure diastereomeric salts (**4″** and **5″**) is first subjected to powder pattern indexing (Dicvol [32]) invoked in the interactive DASH program [23]. In both cases, the indexing algorithms led to definite unit cell parameters of reasonable volume in the crystallographic cell and appropriate cell symmetry. Both **4″** and **5″** salts could be indexed in the monoclinic crystal system and space group, s.g. No. 4 (*P*2_1_), with the help of the build-in ExtSym (Extinction Symbol) program subroutine [33,34], https://www.markvardsen.net/projects/ExtSym/main.html, URL (accessed on 1 February 2026), an algorithm build-in for helping choices of extinction symbol/space group based on calculated probability levels. For the proposed unit cell parameters obtained, see Table 2.

**Figure 9:**
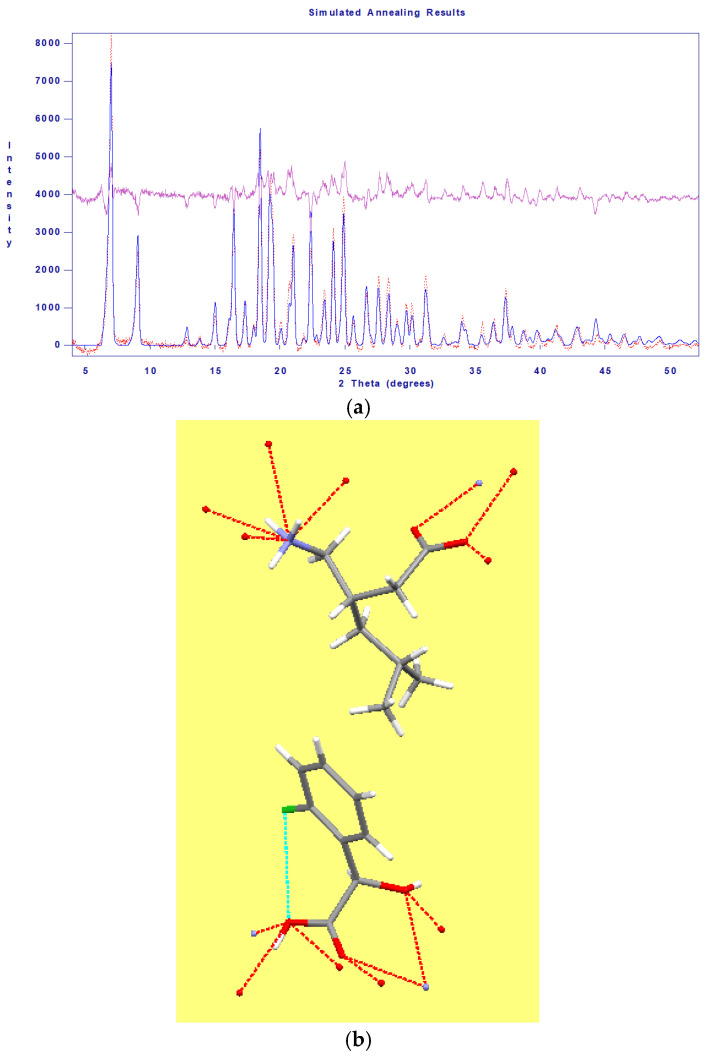
One of the unit cell content-modelling results is shown as both more or less acceptable XRD profile fitting [profile χ^2^= 18.5 after Simulated Annealing, the top curve is the residual difference one] (**a**) and the corresponding molecular conformations in the asymmetric unit (of unit cell s.g. *P*2_1_, Z = 2, Z’ = 1), which exhibits reasonable sophisticated hydrogen bond arrangements around the functional groups (**b**) of (*S*)-pregabalin-(*R*)-2-chloromandelic acid salt (5″) achieved with simulated annealing feature of the DASH program package [23].

Luckily, the estimated unit cell parameters for (*S*)-pregabalin-(*R*)-2-chloromandelic acid salt (**5″**) seem to be quite valid, as the modelling of the unit cell content by simulated annealing (SA) with DASH could lead to quite reasonable molecular conformations without voids via using the atomic distances and bond angles of zwitter ionic (*S*)-pregabalin (CSD code CIDDEZ [36]) and (*R*)-2-chloromandelic acid (CSD code OVIDUT [19]). The best arrangement seems to exhibit at least four hydrogen bonds around the NH_3_^+^ group, three involving the COO^−^ group of (*S*)-pregabalin, and six involving the COOH group, and even two formed with the OH group of (*R*)-2-chloromandelic acid (Figure 9b), resembling to those of sophisticated hydrogen bond systems, which could be observed in the related single-crystal structures of four other related diastereomeric salts, consisting of zwitter ionic pregabalin and unsubstituted mandelic acid enantiomers (CSD codes for single-crystal structures of *S*-pregabalin-*S*-mandelic acid salt are SILFEZ [35], SILFEZ01 [37], while for *R*-*R*, *R*-*S*, and *S*-*R* salts CEWRIJ, CEWQEE, and CEWQOO, respectively, [37]).

Unfortunately, a similarly promising structural content result, arising from the powder XRD profile of the other diastereomeric (*S*)-pregabalin-(*S*)-2-chloromandelic acid salt (4″), precipitating first during the resolution measurements, could not be achieved and could not be drawn by the DASH package. [Note: considering the related pregabalin-unsubstituted mandelic acid resolution system, it would have been worth measuring not only the melting points of the published diastereomeric salts by DSC, but also their enthalpy of fusion, as well, in order to calculate and construct their corresponding ternary phase diagram, where the *S*-*S* diastereomeric salt (mp. 138 °C) is probably less soluble than the *S*-*R* salt (mp. 111 °C) [37] (Supporting Information therein)].

## 3. Materials and Methods

### 3.1. Construction of Triangular Ternary Phase Diagrams of the Resolution Systems

A common quick test for the existence of one or more diastereomeric crystalline phases in the resolution system is dissolving the racemic sample to be optically resolved together with the chosen chiral resolving agent in a stoichiometric (usually 1:1) molar ratio, letting them evaporate to dryness, then checking the final sample’s powder X-ray diffraction (XRD) profile if it is different from those of the starting materials. This test composition point (1:1 molar ratio) sits in the middle of the vertical weight line that bisects the whole triangle diagram (Figure 1 and Figure 2, red crosses). As shown above, it is also worth checking the final dried sample’s powder X-ray diffraction (XRD) profile, whether it is different from those of the similarly prepared diastereomeric salt pair or not.

The representative ternary phase diagram itself might usually be constructed by measuring the melting point and molar enthalpy of fusion via differential scanning calorimetry (DSC) for all the pure existing crystalline phases in the given resolution system of racemic enantiomers and the chosen resolution agent, including their known or possible diastereisomeric salts prepared separately. Calculation of such an estimated triangular melting phase diagram is based on the assumptions of eutectic relation (and validity of simplified Schröder–van Laar equations) among three of the involved crystalline phases pairwise, creating, in this way, several ternary subdiagrams to be joined into a whole one.

### 3.2. Powder X-Ray Diffraction (XRD)

Powder XRD patterns were recorded with a X’pert Pro MPD (PANalytical B.v., Armelo, The Netherlands) multipurpose X-ray diffractometer using Cu Kα_1_ radiation (λ 1.5406 Å) with Ni filter, X’celerator detector, and “zero background” single-crystal silicon or “top-loaded” sample holders in the range of 2θ = 4–44°, with a measuring step size of 0.0167°. The X-ray tube was operating at 40 kV and 30 mA.

### 3.3. Unit Cell Indexing and Structure Modelling Based on Powder XRD Pattern by DASH Program Package

For the purposes of indexing a crystallographic unit cell [32], searching for space group symmetries [33,34], and structural modelling with simulated annealing, originally build-in or invocable program routines of the DASH [23] software package (Release 4.0.0) and a 63 min long profile collection time up to 2θ = 52° were applied.

### 3.4. FT-IR Spectroscopy

Fourier-transform infrared spectra of the solid powdered samples were measured using a PE System 2000 (Perkin Elmer, Shelton, WA, USA) FTIR spectrophotometer in KBr between 500 and 4000 cm^−1^.

### 3.5. Differential Scanning Calorimetry (DSC)

DSC measurements were performed in a DSC 2920 device (TA Instruments Inc., New Castle, DE, USA). The powdered samples (3.5–4 mg) were measured in hermetically sealed Al pans at a heating rate of 10 K/min. A pure indium (In) metal piece was applied for the calibration of temperature and enthalpy, with an empty sealed pan as a reference.

## 4. Conclusions, Assumptions and Limitations

Compressing experiences of resolution trials, based on the formation of diastereomeric salts or co-crystals with different solubilities, into ternary equilibrium melting phase diagrams of the two chiral enantiomers and a resolving agent should include a collection of relevant crystalline phases of the given resolution system and the DSC measurement of their melting points and enthalpy of fusion. Usually, the difference in the observed data of corresponding diastereomeric salt pairs already allows an implication for the solubility relation of the salts during a fractionated crystallization, for a possible result of a resolution test starting from a stoichiometric (e.g., 1:1 molar) mixture of racemic composition with the chosen resolving agent. There is an inverse corroboration between the melting points and solubilities. The higher the melting point is, the smaller the solubility of the salt, and vice versa. An “efficient resolution can be expected, if there are at least 20 K differences between the melting points of the diastereoisomeric salt pair” [7].

Novel construction of a ternary (triangular) phase diagram of *p*- or *o*-chloromandelic acids with chiral 1-cyclohexylethylamines has been carried out. Beyond the expected diastereomeric salt pair, there was an unexpected occurrence of a special diastereomeric ‘double salt’, a mixed salt of the expected salt pair ([16], in both Cases Nos. 1 and 2 (Figure 3 and Figure 4). Unfortunately, in such a case, there is not any initial compositional ratio, to be applied for resolution, which would be in a suitable composition region specifically favoring either of the expected chiral diastereomeric salts. In both studied cases, the usual starting 1:1 molar ratio itself corresponds and leads to the mentioned special double salt containing both enantiomers in equal amounts, i.e., no resolution of *p*- or *o*-chloromandelic acids can be expected with a chiral 1-cyclohexylethylamine as the resolving agent.

Nevertheless, a novel resolution system of racemic *o*-chloromandelic acid with a chiral pregabalin base does not contain any double salt of the expected diastereomeric salt pair, and a large difference (ca. 50 °C) can be observed in the melting points of corresponding diastereomeric salts, so a very asymmetric ternary triangular phase diagram can be constructed (Case No. 3, Figure 6), where the usual starting composition point, the 1:1 molar ratio of racemate to resolving agent, is within a large domain, where the dominating species is the (*S*)-pregabalin-(*S*)-2-chloromandelic acid salt of higher melting point and lower solubility, providing a first precipitate for a successful resolution. Later on, after filtration, from the mother liquor, the less soluble salt, the (*S*)-pregabalin-(*R*)-2-chloromandelic acid salt, also crystalized perfectly. It was so perfectly grown that we have been able to index and model the diastereomeric salts’ unit cell by means of powder X-ray diffraction, i.e., applying both the DASH software package and crystal coordinates coming from former single-crystal X-ray structure determination, resulting in a quite reasonable spatial arrangement (Figure 9b), resembling those of sophisticated hydrogen bond systems, which could be observed in the single-crystal structures of several related diastereomeric salts, consisting of zwitter ionic pregabalin and the unsubstituted mandelic acid enantiomers [35,37].

**Figure 10:**
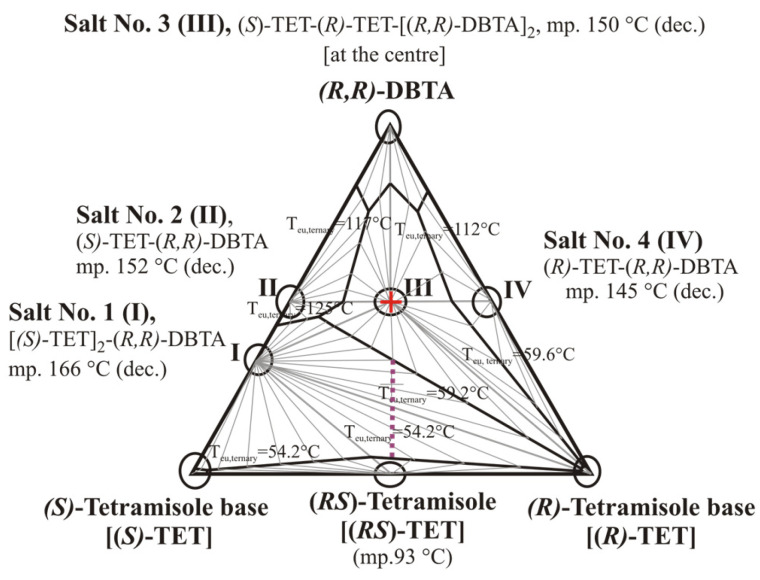
Calculated informative three-component melting phase diagram for system of (*S*)-, (*R*)-tetramisole bases (**TET**), and (*R*,*R*)-*O*,*O*′-dibenzoyltartaric acid (**DBTA**), redrawn from ref. [38] (Figure No. 11 therein, with courtesy). The observed altogether eight solid crystalline components (circled) of eutectic relations, including racemate (*RS*)-tetramisole to be resolved, and four supramolecular salts (**Salt Nos. 1–4**) are marked by circles. **Salt No. 2 (II)** and **Salt No. 4 (IV)** correspond to the expected two diastereomeric salt species, while **Salt No. 3. (III)** is a co-crystallized “double salt” compounds of the two enantiomeric tetramisoles and O,O′-dibenzoyl-(R,R)-tartaric acid (corresponding also to a usual test composition point, 1:1 molar ratio of racemate to resolving agent, indicated by a red cross, as well), meanwhile **Salt No. 1 (I)** is the real key compound of successful resolution, which can be formed between chiral (*S*)-tetramisole base **(*S*)-TET** and (*R*,*R*)-dibenzoyltartaric acid (**DBTA**) in 2:1 molar ratio. This latter diastereomeric crystalline salt is the dominating solid phase in a large compositional domain, in which the dotted lilac line sequence corresponds to potential variety of promising mixing ratios between crystalline racemate (***rac*-TET**) base and the chiral **DBTA** to achieve a successful resolution.

Anyhow, an existence of a double salt between the two diastereomeric salts with a 1:1 molar ratio of base to acid might not necessarily result in an ‘impossible resolution mission’ of the given resolution system. There might be some exceptions, especially where there is an opportunity for the formation of salts with different stoichiometrics, e.g., of a diastereomeric salt with 2:1 molar ratio of the base to acid with two acidic groups, as it was found in the case of racemic tetramisole free base and *O*,*O*’-dibenzoil-(*R*,*R*)-tartaric acid [38]. Actually, successful resolution could be achieved by applying the dibasic dibenzoyl tartaric acid as a resolving agent, when added in less than one-half molar equivalent amount compared to the tetramisole racemic base, through the formation of an unexpected individual salt with a 2:1 = base/acid molar ratio in the ternary system (Figure 10, Salt No. 1 (I)) [38].

All of the mentioned cases, altogether, show the high importance of one of the limitations and assumptions, namely that all of the solid crystalline phases (salts) present/existing in the system should be taken into account when a relevant ternary (triangular) phase diagram of a given resolution system is constructed, beyond the basic assumption that all of the crystalline phases are in ideal eutectic relations. Luckily, if the initial racemic composition to be resolved is a conglomerate of chiral enantiomeric crystals, then, usually, a successful resolution can be achieved by applying seeding crystals of the required enantiomeric species (‘preferential crystallization’, also known as ‘crystallization by entrainment’) [39,40]. The third group of racemic compositions, the so-called pseudo-racemates (solid solutions), is luckily rare, and the resolution process might require special considerations [1].

## Figures and Tables

**Table 1:** Melting point and enthalpy of fusion of solids in the systems of *p*- and *o*-chloromandelic acids with (*S*)-1-cyclohexylethylamine, applied in the calculations of binary and ternary phase diagrams using the simplified Schröder–van Laar * (or Prigogine–Defay **) equations.

Solids	Compound Label	Melting Point (T^f^_i_ Peak Temperature °C/K)	Enthalpy of Fusion, ΔH_i_ (J/mol)	Source of DSC Data (Ref.)
(*S*)-4-chloromandelic acid	(*S*)-4ClMA, **1**	123.2/396.3	21,850	[15]
(*R*)-4-chloromandelic acid	(*R*)-4ClMA, **3**	123/396.3	21,850	[assumed to be the same as that of *S*)-enantiomer]
*rac*-4-chloromandelic acid	*rac*-4ClMA, **2**	122.4/395.4	50,418	[15]
*S*(*R*)-*S*(*R*)-salt of 4ClMA with CHEA	(*S/R*)-CHEA- -(*S/R*)-4ClMA, **4**	161/434.1	31,585	[16] Data read-outs from the ESI
*S*-*R*-salt of 4ClMA with CHEA	(*S*)-CHEA- -(*R*)-4ClMA, **6**	157/430.1	26,736	[16] Data read-outs from the ESI
(*S*)_2_-(*S&R*)-double salt of 4ClMA	[(*S*)-CHEA]_2_- -(*R*&*S*)-4ClMA, **5**	142/415.1	65,680	[16] Data read-outs from the ESI
(*S*)-2-chloromandelic acid	(*S*)-2ClMA, **1′**	120/393.1	24,033	[15]
(*R*)-2-chloromandelic acid	(*R*)-2ClMA, **3′**	120/393.1	24,033	[assumed to be the same as that of S)-enantiomer]
*rac*-2-chloromandelic acid	(*rac*-2ClMA, **2′**	89/362.1	43,076	[15]
*S*-*S*-salt of 2ClMA with CHEA	(*S*)-CHEA-(*S*)- -2ClMA, **4′**	140/413.1	13,200	[16] Data read-outs from the ESI
*S*-*R*-salt of 2ClMA with CHEA	(*S/R*)-CHEA- -(*R*/*S*)-2ClMA, **6′**	127.5/400.6	13,714	[16] Data read-outs from the ESI
(*S*)_2_-(*S&R*)-double salt of 2ClMA	[(*S*)-CHEA]_2_- (*R*&*S*)-2ClMA, **5′**	151.5/424.6	54,832	[16] Data read-outs from the ESI

* Liquidus curves according to Schröder-van Laar’s equation: ln *x*_i_ = Δ*H*_i_/*R* (1/*T*^f^_i_ − 1/*T*^f^); (*i*= 1, 2, or 3); Σ*x*_i_ = 1. ** Liquidus curves according to Prigogine-Defay’s equation: when *x*_eu_ ≤ *x*_S_ ≤ 1 − *x*_eu_ then ln [4*x*_S_(1 − *x*_S_)] = Δ*H*_RS_/*R* (1/*T*^f^_RS_ − 1/*T*^f^).

**Table 2:** Guesses for crystallographic unit cell parameters of (*S*)-pregabalin-(*R*)-2-chloromandelic acid (**5″**) and (*S*)-pregabalin-(*S*)-2-chloromandelic acid (**4″**), estimated from the measured powder XRD patterns by powder pattern indexing [32] using interactive DASH program [23] in comparison with room temperature single crystal unit cell data of (*S)*-pregabalin-(*S*)-2-mandelic acid [35].

Crystallographic Unit Cell Parameters	Prepared Pure (*S*)-Pregabalin-(*R*)-2-ClMA (5″, rt.) [This Work]	Recrystallized Precipitation of (*S*)-Pregabalin-(*S*)-2-ClMA (4″, rt.) [This Work]	SILFEZ (*S*)-Pregabalin-(*S*)- -2-Mandelic Acid (rt.) [35]
Chemical formula	C_8_H_17_NO_2_· ·C_8_H_7_ClO_3_= =C_16_H_24_ClNO_5_	C_8_H_17_NO_2_ ·C_8_H_7_ClO_3_= =C_16_H_24_ClNO_5_	C_8_H_17_NO_2_· ·C_8_H_8_O_3_= =C_16_H_25_NO_5_
Crystal system	monoclinic	monoclinic	monoclinic
Space group	*P*2_1_ (No. 4)	*P*2_1_ (No. 4)	*P*2_1_ (No. 4)
a (Å)	13.56	14.19	6.292 (1)
b (Å)	6.742	6.55128	27.423 (6)
c (Å)	10.42	10.12	10.009 (2)
α (°)	90	90	90
β (°)	106.6	102.0	90.84 (3)
γ (°)	90	90	90
V (Å^3^)	912.5	920.1	1726.877
Z/Z′ (No of formula units)	2/1	2/1	4/2
V_m_ (Volume per formula unit) (Å^3^)	456.2545	460.0605	431.719
Zero-point shift (°)	0.2724	0.2947	
Zmatrices	CIDDEZ_1 + OVIDUT_1	CIDDEZ_1 + GASCEK_1	
Degrees of freedom	19 = 12 + 7 (torsional)	19 = 12 + 7 (torsional)	
Pauli refinement (χ^2^)	3.852	10.30	
Profile χ^2^ after SA *	18.75	221.5	

* For definition of profile χ^2^ after Simulated Annealing (SA), see world wide web, at link to http://github.com/ccdc-opensource/dash/wiki/FiguresOfMerit [URL (accessed on 1 February 2026)].

## Data Availability

Data are available from the author upon personal request.
